# The Notion of Coopetition-Based Open-Innovation in Business Practices: A Model to Accelerate Firm Performance

**DOI:** 10.3389/fpsyg.2022.931623

**Published:** 2022-08-11

**Authors:** Andrianarivo Andriandafiarisoa Ralison Ny Avotra, Ye Chengang, Kmashi Said Mohamed Said, Chunhong Chu, Li Xiang

**Affiliations:** ^1^Business School, Zhejiang Wanli University, Ningbo, China; ^2^Business School, University of International Business and Economics, Beijing, China; ^3^College of Economics and Management, Zhejiang Normal University, Jinhua, China; ^4^School of Accounting, Shandong Technology and Business University, Yantai, China; ^5^Business School, Huanggang Normal University, Hubei, China

**Keywords:** firm performance, open innovation, decision making, coopetition, trust and dependency

## Abstract

In today’s competitive business market, firms that collaborate on a coopetition basis face obstacles in the form of decision-making, dependency, and trust in their competitor partners. This current study is the only one that has examined the relationship between coopetition and firm performance; yet, this relationship appears to be unclear due to the impact of trust and dependency on coopetition. This study investigates the impact of coopetition on firm performance by examining the mediating effects of decision-making and open innovation on firm performance. There are 230 sets of data that were collected from the employees of Chinese small- and medium-sized enterprises through the survey method, and the data were analyzed using Partial Least Square-Structural Equation Modeling. The findings of this study indicated that open innovation has a significant mediation effect between coopetition and firm performance, and that decision-making is also an important mediating effect in bridging the relationship between coopetition and firm performance. By considering these mediators, the findings revealed that the coopetition has a significant impact on firm performance through decision-making and has a significant effect on firm performance through increasing open innovation. The findings also revealed that decision-making played a significant role in mediating the relationship between coopetition and firm performance, which in turn specified a statistically significant positive relationship with decision-making that mediated a positive relation. According to the findings of this research, modern business firms should recognize the relevance of coopetition-based open innovation in their business processes to increase their overall performance. This study is significant because it provides a game-changing strategy for the management of businesses.

## Introduction

In this decade, businesses are facing different problems related to performance and the competition in the target market due to the role of management and the stakeholders. In this regard, to improve the business performance, the term coopetition is introduced into the business sector: this is where organizations join each other and work progressively (Dyanasari et al., 2021). However, the role of trust and dependency is important in this kind of business relationship because when different businesses join together to progress and improve their business performance, the roles of open innovation and decision-making are important for firm performance (Venesz et al., 2022). In business performance, trust refers to believing in each other and working comfortably to increase open innovation and improve the business performance (Mathafena and Msimango-Galawe, 2022). At the same time, dependency refers to the reliance of one business over the other to utilize the resources and progress in a better way, and this dependency could be of any type: resources, talents, or the supply chain (Dinh et al., 2022). Coopetition in business refers to the strategy of business planning, where different businesses join together to improve their business performance and develop a common strategy for the competitive businesses (Aydiner et al., 2019). This strategy is widely used by competitors in markets to become market leaders and compete against other similar businesses. Decision-making refers to taking actions based on qualitative or quantitative data; it could also manifest in the form of intuition (Fernández-Portillo et al., 2022). Open innovation refers to the sharing of ideas with different business firms, targeting market innovation to improve business performance, and providing information to the consumers (Shaikh and Randhawa, 2022). Finally, the performance of any firm refers to the strategies and activities being carried out to improve the transaction of any business (Chaudhary et al., 2022). These variables are important for the performance of firm, but most firms do not consider these for the improvement of the performance of their business (Venesz et al., 2022).

A significant number of studies have been carried out to uncover the factors that lead to decreased firm performance over the decades. In coopetition research, several studies have demonstrated that factors such as resources and capabilities are all contributing to the decline in the performance of firms (Shmeleva et al., 2021). A variety of analyses of firm performance have been conducted concerning competition activities, and the results have shown that smaller firms that collaborate with their competing firms are more likely to perform better than those that do not use their competing companies’ resources and expertise (Guertler and Sick, 2021; Lam et al., 2021; Qureshi et al., 2021). Various researchers are looking at ways to improve the performance of businesses. Strategic alliances, wages, net income per employee, economic globalization, seasoned equity offering, quality management, and incentive design were identified as predictors of coopetition (Hameed et al., 2021). Many studies on firm performance have been conducted in the past, but there is one aspect that has been neglected or given less attention than it deserves (Nawaz et al., 2022). Coopetition is one of the most important factors that have a direct impact on the performance of a firm (Tokede et al., 2022). These findings may be very beneficial to firms. As an extra point of reference, several studies have looked into the relationship between coopetition and firm performance. The researchers employed a contemporary working setting to assess the dimensions of coopetition, which is why the researchers selected this survey method. The link between coopetition and firm performance has received less attention in the past (Venesz et al., 2022). More importantly, this relationship has not been investigated among the small- and medium-sized enterprises (SMEs) of China. The growth of Chinese SMEs is significantly increasing. Each year, the number of SMEs in China is increasing. There are about five million SMEs in China, representing at least a 10% year-over-year growth rate; however, the performance of open innovation is low in these SMEs. Therefore, the scope of this study is grounded on the Chinese SMEs in which the phenomenon of coopetition and open innovation is investigated.

An additional goal of this study is to investigate whether it is necessary to incorporate decision-making and open innovation variables as mediating variables to resolve the inconsistent relationship between coopetition and firm performance. As mediating variables in our study, we place a strong emphasis on decision-making and open innovation since earlier research has indicated that these two elements are key determinants of firm performance (Venesz et al., 2022). In previous major studies that have been done on coopetition and firm performance, the ambiguous missing link has been identified, and many studies important to this area have advised that decision-making and open innovation must be included as mediating variables in future research (Hervas-Oliver et al., 2021; Roh et al., 2021; Venesz et al., 2022). In addition, decision-making and open innovation were employed as mediating variables in the present research to reconcile the conflict between the independent and dependent variables. Related to the link between coopetition and firm performance, this has received less attention in the past. Therefore, it is probable that the results of this study do not effectively represent the mediating influence of decision-making and open innovation on firm performance as a consequence of this. The objective of this study is to highlight the role of open innovation in the firm performance related to coopetition business where different firms join hands to improve the business performance, achieve the target market, and develop a competitive advantage for the other business. In this regard, this study is designed to analyze the role of coopetition with the mediation of open innovation and decision-making and to improve business performance. Also, the study aims to provide a comprehensive structure for those firms that are joining each other for competing businesses and improving the business performance while utilizing the resources and talent. This study provides detailed information on the coopetition to improve the business performance and the role of trust and dependency in coopetition.

**FIGURE 1:**
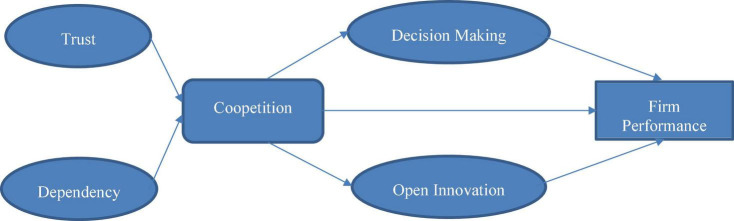
Conceptual framework.

Moreover, this study highlights how open innovation mediates the relationship between coopetition and business performance because in the modern time, the different business firms are using coopetition to improve the business performance. Also, this study demonstrates the mediating role of open innovation in the relationship between coopetition and business performance (Venesz et al., 2022). Indeed, it is observed that for business performance, coopetition is necessary with the key role of open innovation and decision-making. This study has theoretical as well as practical implications for the improvement of business performance related to coopetition between different businesses and providing a relationship between variables (see Figure 1). To begin with, it highlights the important role of trust and dependency in the coopetition of business because, it is important for the business firms to build trust and dependency while working jointly to improve the business performance. At the same time, this study addresses a theoretical gap that was not discussed earlier by any study in the relationship of business performance, and coopetition; the role of open innovation and decision-making, is crucial to develop one table strategy and get progress in the target market. On the other hand, this study provides managerial implications that with the help of coopetition based on open innovation and decision-making could improve the business performance, and it could help businesses compete with the other businesses and utilize the same resources to work progressively.

## Review of Literature and Hypothesis Development

### Role of Trust in Business Coopetition

The modern business firms are working jointly using coopetition to improve business performance while working with different stakeholders and in the different target markets. However, this coopetition is no easy task, because diverse people are working with joint effort, and in this regard, cultural and social clashes are common (Marilang, Amiruddin and Syatar, 2021). Similarly, to improve the social clashes, the role of trust is necessary between the partners when they are working with coopetition (Dar et al., 2022). It is noted that the business firms that use coopetition do not have an issue with trust; as a result, the performances of these kinds of businesses are improved. On the other hand for the business firms that are joined to work mutually based on trust, competition between them fails, and they starts to separate. Trust is the unique key to joining hands with other businesses to improve their relationship for the long term, and improve the business performance at the same time.

***H1:*** *Trust has an important role in business coopetition*.

### Role of Dependency in Business Coopetition

Modern businesses are working on being dependent on other businesses to be sustainable and gain a competitive advantage (Pattinson et al., 2022). In this regard, for the coopetition businesses working with a dependency model, it is observed that when a business has a deficiency in the supply chain, but its partner has efficiency in the supply chain, then both businesses join to provide an effective supply chain for both of them to improve their business performance. However, on the other hand, according to Aydiner et al. (2019), when the businesses are not dependent on each other but are working in coopetition, then it is difficult for these businesses to be sustained in the market for the long term. Besides, business coopetition is not limited to increasing profit and revenue; it also addresses the issues of a partner business, such as difficulties during the marketing, operation, or the production process (Tokede et al., 2022). However, It is also noted that the performance of any business is affected when the decisions are not taken on time and businesses then loose market value (Hair et al., 2019; Giantari et al., 2022).

***H2:*** *Dependency has an important role in business coopetition*.

### Role of Coopetition in Business Performance

Indeed, business coopetition is an emerging concept that seeks to expand the market share and achieve a joint competitive advantage by sharing ideas, talents, and resources to satisfy the market. Furthermore, the businesses that are unable to perform all the activities in the particular market, provide satisfaction to the consumer, and expand the market share, these business needs to work in coopetition, to enhance the experience and talent, and utilize all in a positive way, to attract the consumer in the target market (Shmeleva et al., 2021). On the other hand, the businesses that have a deficiency in supply chain or any other operational activity and are not in coopetition to increase the business risk their competitors gaining access to their potential consumers. In this way, business coopetition provides opportunities for the businesses to have sustainable development, utilize limited resources to increase their business performance over time, and capture a big share in the market (Dinh et al., 2022; Giantari et al., 2022).

***H3:*** *Coopetition has an important role in increasing business performance*.

### Role of Coopetition in Decision-Making

There are several reasons to believe that sports and physical activities provide a good environment in which to develop these abilities. Numerous youngsters already engage in sports activities, whether individually or in groups, structured or unstructured, but these settings provide numerous chances to grow essential skills such as teamwork as well as collaboration, compassion and pro-social behavior, making plans, and solving problems. According to the Sports and Fitness Industry Association, which counts participation in 120 sports, recreation, and fitness activities in the United States, more than 70% of children aged 6 to 12 years participate in team sports at least 1 day each year (Hurd and Deutsch, 2017). Academic development in school necessitates mastery of certain abilities, such as social and academic work. Coopetition in businesses helps to achieve the right decision for increasing the firm performance because it is noted that when the individual business entity is working separately, and there is no coopetition with other businesses, then it becomes difficult for that firm to get the right decision, due to the lack of resources and external information (Dyanasari et al., 2021; Hervas-Oliver et al., 2021; Roh et al., 2021). However, businesses that work using coopetition increase their performances to provide comprehensive strategies and expand their market shares while making the right decisions. According to Khattak and Saiti (2021), the business performance and the right decisions are improved when organizations joins hands and expand their ideas and open up for innovation performance to increase the market share; the right decision-making is then important to make progress in the target market.

***H4:*** *There is an important role of coopetition in business.*

### Role of Coopetition in Open Innovation

It is a fact that when businesses are working with coopetition and organizations are sharing ideas and talent to increase their business performance and capture the target market, open innovation is affected because of the sharing of ideas and external knowledge. In modern times, businesses are joining each other due to open innovation because open innovation is key to success in the target market, as it provides information to the consumers and competitors about the idea behind the product or service and removes all barriers in the way of progress (Marilang, Amiruddin and Syatar, 2021). However, the business entities need to develop trust and dependency to increase the open innovation when they are in coopetition to expand the market share and increase the business performance. On the one hand, open innovation is found when businesses are working and sharing ideas, and as result, the market share of such kinds of businesses increases due to their unique strategy for open innovation and sharing of the internal and external information with the firms (Fauzi, 2021). On the other hand, according to Mulyanah and Saadati (2021), it is also noted that the business entities that work differently and do not share ideas and internal or external knowledge with the other business firms are not trustworthy, and coopetition with them is not acceptable because they lack the idea of open innovation and merely wish to increase their market share to build a competitive advantage rather than sharing ideas with other businesses to work jointly in the target market.

***H5:*** *Coopetition has a key role for open innovation in businesses.*

### Role of Business Decision-Making in Business Performance

As for as the performance of any business is concerned, the role of the right decision is important; correct decision-making allows businesses to work in a comfortable environment and take advantage of all opportunities to target and generate revenue (Avotra et al., 2021). However, it is a fact that most business entities are not so competitive that they could analyze the situation, and the management of such businesses aims to make the right decisions, provide a comprehensive plan, and increase the market share for better clarity. In this way, according to Hameed et al. (2021), it is noted that when businesses are working in coopetition and the firms are sharing ideas jointly, the right decisions are being made to increase the business performance so that the business performance increases. According to Chaudhary et al. (2022), the performance of any business is directly dependent on the relationship between the management and the decision-making process because businesses expect that the management should make the right decision at the right time to increase market shares. Correct decision-making is the key to success, and it helps to build the competitive advantage by enhancing the front performance and making the business a top leader by sharing ideas in coopetition and improving external knowledge to facilitate correct decision-making (Hervas-Oliver et al., 2021).

***H6:*** *There is the important role of decision-making in firm performance*.

### Role of Open Innovation in Business Firm Performance

Open innovation refers to the sharing of ideas and external knowledge to increase firm performance (Hervas-Oliver et al., 2021). In this way, open innovation has become a key factor in increasing business performance in the modern time, because this idea is still developing and the business entities are rising to capture a big share in the market. On the one hand, some businesses are considering that open innovation is best for the businesses because when the ideas are shared, the innovation in the market is for the benefit of consumers; at the same time, the consumers are provided with information about the products and services. Therefore, the performance of the business entity is increased due to open innovation. Similarly, on the other hand, some businesses believe open innovation should not be done because it is not best for the target market as the competitor gains the advantage; they try to produce the product or service using a similar idea. In this way, the integrity of business performance is challenging, but these business entities, when working jointly or as a separate entity, want to maintain the large market share and increase the business performance (Shaikh and Randhawa, 2022). In the same way, the business entities that are working in competition aim to increase the business performance while working jointly with the competitors and providing them with sufficient help to reduce the weaknesses of the business; in such kinds of business entity, the role of open innovation is important because the firm’s performance is dependent on open innovation. Venesz et al. (2022) state that when several businesses that are working with the idea of open innovation and their performance is increased over time by sharing ideas, values, talents, and knowledge about the production, efficiency, and effectiveness have become part of the business performance the performance of the first kind of businesses is increased.

***H7:*** *Open innovation has an important role in firm performance.*

### Mediating the Role of Decision-Making and Open Innovation Between Coopetition and Business Performance

Decision-making is an important factor related to coopetition between the businesses and the business entities that are working to increase the current performance (Jamal Ali and Anwar, 2021). Indeed, for the business entities that are working jointly, the strategies are being framed on a single table and the ideas are shared with the other firms, and the role of management is important to provide a single way to increase the business performance. In this regard, according to Shaikh and Randhawa (2022), the role of decision-making is important because it mediates the relationship between coopetition and business performance, as it is understood that the role of business performance is directly dependent on the decision-making; the more decisions are right, the more the result are in favor of the business entities, and in this regard, the performance of these businesses would be increased automatically. However, the performance of any business is affected when decisions are not taken in time and businesses then loose market value (Galanti et al., 2020). Furthermore, decision-making is a crucial factor that is important to understand, but should not be taken for granted, as it plays a key role in mediating the relationship between business performance and coopetition. Also, Latheef et al. (2021) stated that coopetition without making a timely decision for any business activity would be failed, and in such cases, the performance of these businesses decreases. When the businesses that practice correct decision-making work in coopetition and utilize all the resources of the competitors in an effective and efficient manner, business performance is increased because they have taken the efficiency and effectiveness of the business activity from the production to the market. Open innovation also mediates the relationship between business performance and coopetition because when businesses work jointly to increase the market share then their coopetition is important to increasing the sharing of external knowledge and internal information to the joint business firms, and in this regard, open innovation is granted. The more open innovation a business has, the more effective it is, and in this way, the market share is expanded and the performance of business entities is increased automatically (Almeida, 2021).

***H8:*** *Decision-making has a mediating role in the relationship between coopetition and business performance.*

***H9:*** *Open innovation has a mediating role in the relationship between business performance and coopetition.*

The following conceptual model (Figure 1) has been formed based on the above literature and the hypothesis.

## Methodology

### Questionnaire Preparation

The questionnaire for this study was prepared with careful consideration, and it was divided into two sections; in the first section, the information about the demographics of respondents were collected, and in the second section, the scale items for each variable, were provided. In this regard, the scale items for variables; trust, coopetition, dependency, and open innovation have been taken from the study of Hills and Eraso (2021). However, the scale item related to decision-making and firm performance was taken from the study of Aydiner et al. (2019). Also, the questionnaire contained no ambiguous questions but the scale items for the 5-point Likert scale were adopted for the questionnaire.

### Variable Measures

The scale items taken to prepare the questionnaire were carefully considered because the purpose of these items is to understand the role of cooperation-based open innovation in business performance. In this regard, the scale items of coopetition were selected to understand the role of a joint venture by different business firms to check the experience of the staff and the managers. In the same way, the scale items taken for the measurement of open innovation are used in this study to understand the role of open innovation in firm performance and how it could be increased. Furthermore, it was aimed that the questionnaire should be discriminant from the perspective of variables because it was decided that no variable should alternate in the scale items and there should be a clear difference between them. Also, on the other hand, the questionnaire was designed to measure the variables in detail.

### Data Collection Method

For this study, the data were collected from the different managerial level staff of Chinese manufacturing SMEs because managerial employees have better ideas related to the open innovation and coopetition. Furthermore, data were collected to understand the relationship between the variables and their impersonal feedback on the questionnaire. To begin with, the questionnaire was delivered to the respondents to get their responses. Also, their personal information provided to them would not be shared with any third party and is only useful for this research study. In the same way, the purpose of research was explained to them to get a response positively and make sure that they are contributing to the worth of this research. In this regard, the questionnaire was collected after getting a response from the respondents, and the respondents were appreciated for their precious time and response. A total of 500 questionnaires are distributed and 230 are returned, and these were used in data analysis.

## Findings

This section of the study contains the findings based on the data collected by the questionnaire. In this way, the Smart-PLS was used to analyze the data in this study. Smart-PLS is a useful tool to analyze convergent and discriminant validity (Xiaolong et al., 2021; Yingfei et al., 2021). However, the tool was used to obtain the data related to the factor loadings, the composite reliability (CR), and the average variance extracted (AVE) value. Also, the PLS algorithms and bootstrapping were used to check the results.

### Convergent Validity

**FIGURE 2:**
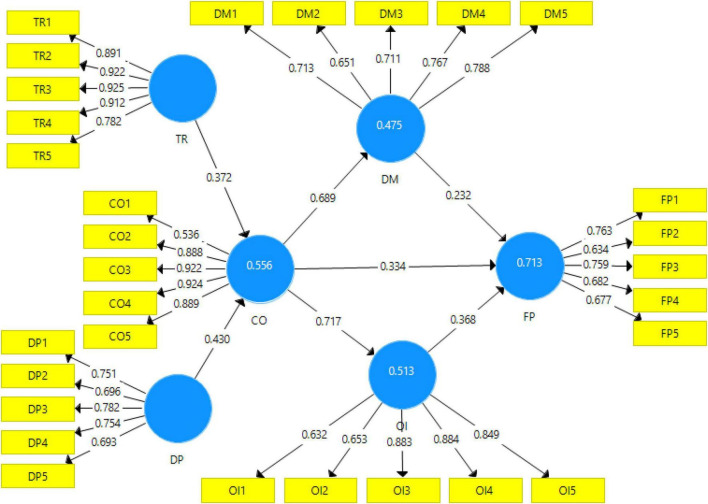
Output of measurement model algorithm. “CO, coopetition; DP, dependency; DM, decision-making; TR, trust; OI, open innovation; and FP, firm performance.”

**FIGURE 3:**
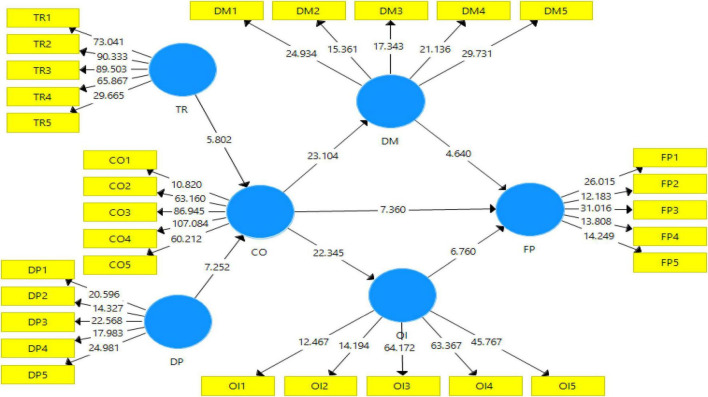
Output of structural model bootstrapping. “CO, coopetition; DP, dependency; DM, decision-making; TR, trust; OI, open innovation, and FP, firm performance.”

In this section of the study, the factor loadings were analyzed and displayed in the measurement and structural model (see Figures 2, 3). To begin with, the factor loadings were analyzed and the factor loadings for all the scales except for one were greater than 0.6. In this regard, the CR value for all the variables is greater than the recommended value of 0.70 by Hair et al. (2019). On the other hand, the AVE value for all the variables is greater than the recommended value of 0.50. Similarly, the value of Cronbach alpha for all the variables is greater than the value of 0.7 which is recommended and accepted for reliability (see Table 1).

**TABLE 1:** Factor loadings, reliabilities, and AVE.

Variables		Items	Loadings	Alpha	CR	AVE
Trust	TR1	Our cooperation partner has always been evenhanded in its negotiations with us	0.891	0.932	0.949	0.789
	TR2	Our cooperation partner is trustworthy.	0.922			
	TR3	Our partners related to cooperation keeps up with their promises.	0.925			
	TR4	Our partners trust us.	0.912			
	TR5	Our partners are innovative.	0.782			
Dependency	DP1	Our partners related to cooperation has a solid comparative negotiating position with us.	0.536	0.798	0.855	0.542
	DP2	Our partners related to cooperation observe minor differences among our products as well as those of our competitors.	0.888			
	DP3	We must obey with various demands of our partners, even if they seem unsuitable.	0.922			
	DP4	Our partners are dependent on us.	0.924			
	DP5	We are dependent on our partners.	0.889			
Coopetition	CO1	We are in close coopetition with our partners	0.536	0.889	0.924	0.714
	CO2	We collaborate with competitors to achieve a common goal.	0.888			
	CO3	An active coopetition with our collaborators is important to us	0.922			
	CO4	Coopetition provides a competitive advantage.	0.924			
	CO5	Coopetition increases a firm’s performance.	0.889			
Decision making	DM1	Right decisions are improving firm performance.	0.713	0.779	0.848	0.529
	DM2	Right decisions are impossible.	0.651			
	DM3	Coopetition helps in the right decisions.	0.711			
	DM4	The joint effort helps in the right decisions.	0.767			
	DM5	Coopetition helps for the right decisions.	0.788			
Open innovation	OI1	New ideas are always welcomed in our alliance.	0.632	0.843	0.889	0.622
	OI2	Communication between internal and external partners occurs without problems which increase OI	0.653			
	OI3	The degree of knowledge shared is sufficient to enhance openness.	0.883			
	OI4	All the partners know exactly which knowledge is needed for OI	0.884			
	OI5	All partners are willing to learn from the experiences in this alliance.	0.849			
Firm performance	FP1	Firm performance is dependent on the right decision.	0.763	0.748	0.831	0.497
	FP2	Firm performance is dependent on open innovation.	0.634			
	FP3	Firm performance is dependent on coopetition.	0.759			
	FP4	Trust is necessary for firm performance.	0.682			
	FP5	Management role is important in firm performance.	0.677			

### Discriminant Validity

In this section of the study, the discriminant validity was checked using Smart-PLS as recommended by modern studies (see Table 2). Furthermore, the HTMT method was used to determine the values for each variable because it is used for modern research (Hao et al., 2020; Nawaz et al., 2021). In this regard, the value of all the variables was less than the recommended 0.90. This shows that there is a clear distinction between the scale items of the variables and they are not similar to each other.

**TABLE 2:** Discriminant validity (HTMT).

	CO	DP	DM	FP	OI	TR
CO						
DP	0.779					
DM	0.701	0.815				
FP	0.712	0.749	0.727			
OI	0.716	0.798	0.894	0.857		
TR	0.752	0.771	0.689	0.775	0.779	

*CO, coopetition; DP, dependency; DM, decision-making; TR, trust; OI, open innovation; and FP, firm performance.*

### The PLS-SMEs Model

This section of the study contains the hypothesis test to analyze their significance or insignificance shown in Table 3. H1 was tested to check its significance, and according to the results, TR has a significant effect on CO (β = 0.372, *t* = 5.980, *p* = 0.000) and H1 is supported. H2 was tested to check its significance, and according to the results, DP has a significant effect on CO (β = 0.430, *t* = 7.319, *p* = 0.000) and H2 is supported. H3 was tested to check its significance, and according to the results, CO has a significant effect on DM (β = 0.689, *t* = 23.558, *p* < 0.000) and H3 is supported. H4 was tested to check its significance, and according to the results, CO has a significant effect on OI (β = 0.717, *t* = 23.406, *p* < 0.000) and H4 is supported. H5 was tested to check its significance, and according to the results, CO has a significant effect on FP (β = 0.334, *t* = 7.115, *p* < 0.000) and H5 is supported. H6 was tested to check its significance, and according to the results, DM has a significant effect on FP (β = 0.232, *t* = 4.504, *p* = 0.000) and H6 is supported. H7 was tested to check its significance, and according to the results, OI has a significant effect on FP (β = 0.368, *t* = 6.855, *p* = 0.000) and H7 is supported.

**TABLE 3:** Direct effects.

Hypotheses	B	(STDEV)	*T*-Statistics	*P*-values	Decision
H1. TR ->CO	0.372	0.062	5.980	0.000	Supported
H2. DP -> CO	0.430	0.059	7.319	0.000	Supported
H3. CO -> DM	0.689	0.029	23.558	0.000	Supported
H4. CO -> OI	0.717	0.031	23.406	0.000	Supported
H5. CO -> FP	0.334	0.047	7.115	0.000	Supported
H6. DM -> FP	0.232	0.052	4.504	0.000	Supported
H7. OI -> FP	0.368	0.054	6.855	0.000	Supported

*CO, coopetition; DP, dependency; DM, decision-making; TR, trust; OI, open innovation; and FP, firm performance.*

### Indirect Effect Results

In this section of the study, the mediation or indirect effect was measured, and according to the results, OI mediates the relationship between CO and FP (β = 0.263, *t* = 6.725, *p* = 0.000), hence H8 is supported. Similarly, according to the results, DM mediates the relationship between CO and FP (β = 0.160, *t* = 4.301, *p* = 0.000), therefore, H9 is supported (see Table 4).

**TABLE 4:** Indirect effects.

Mediators	B	(STDEV)	*T*-Statistics	*P*-values	Decision
H8. CO -> OI -> FP	0.263	0.039	6.725	0.000	Mediation
H9. CO -> DM -> FP	0.160	0.037	4.301	0.000	Mediation

*CO, coopetition; DP, dependency; DM, decision-making; TR, trust; OI, open innovation; and FP, firm performance.*

## Discussion

In recent years, cooperative action has evolved into a complex, multi-dimensional, multileveled, and interconnected phenomenon. To properly assess coopetition research, a comprehensive, all-encompassing approach is required, since the contemporaneous contact of cooperative and competitive activities influence various aspects in the interaction and execution process, as described by Brasoveanu et al. (2020). Overuse of coopetition may result in extortion, loss of concentration, diluting of comparative advantages, and poor performance. Because of this, organizations must carefully choose an ideal amount of coopetition to avoid risks and examine the elements that might impact the link between coopetition and firm performance (Aydiner et al., 2019).

The results of H1, H2, and H3 show that there is an important role of trust and dependency in the coopetition of businesses to attain a competitive advantage in the target market. In the same way, it is observed that the role of coopetition is important in decision-making. Indeed, the firms that are working on the coopetition model, they seek decisions that are made with joint effort and shared information that helps to increase the strength of good decisions for the business performance. These decisions are not only limited to increasing the business performance but also play a key role in improving the relationship between the business entities. The results of H4 and H5 show that there is an important role of cooperation in open innovation and firm performance. No doubt, the performance of any firm is dependent on open innovation and cooperation because the business is working in the right direction to enhance the capabilities to target the market and get potential consumers. In this regard, the role of coopetition is important for providing opportunities for business firms. In this way, the performance of firms could be easily increased with joint effort from business entities.

The results of H6 and H7 show that for the business performance, the role of decision-making and open innovation is important because the business firm is dependent on open innovation and supported by the right decision-making; in this way, the performance of business would be increased in the target market. However, joint efforts should be done to provide the shared knowledge and the right decision-making for the open innovation to increase the performance of any business firm in the target market. The results of H8 show that there is a mediating role of open innovation in the relationship between coopetition and firm performance. Indeed, it is a fact that, due to the open innovation with the help of coopetition, the performance of any business can be increased in an understanding and reasonable way. Also, on the other hand, the results of H9 show that there is an important mediating role in decision-making between the relationship between coopetition and firm performance. In this regard, it is noted that the performance of any firm is directly responsible for the factors that are decision-making and coopetition. Therefore, it is a fact that the more coopetition a business has and the more right decisions it makes, the more the performance of the business is increased.

This is the first time that such a unified viewpoint has been produced, and it offers essential direction for both theoretical and empirical discussions as well as a reference point for future investigations in the field. The ability to situate the study and determine which factors are linked to it helps researches further their research. To find benefits, impediments, and possibilities for future study, it is beneficial to do this exercise first. At the same time, research into better identifying the main relationships must be considered to effectively manage coopetition in practice.

## Implementation

### Theoretical Implications

This study has particular implications because a limited number of studies have discussed coopetition in relation to firm performance. However, the mediating role of open innovation from coopetition to firm performance will enhance the performance of future businesses, if the strategies would be made on the results of this study. However, on the other hand, the role of decision-making as a mediator between coopetition and firm performance is important, and this mediating effect was not discussed earlier by any study. In this way, this study has significant implications for the provision of comprehensive literature for future studies to understand the relationship between the variable found in a theoretical framework and at the same time improve business performance when companies are working in cooperation.

### Practical Implications

This study has practical implications: future businesses should understand the role of decision-making and open innovation in coopetition because when business firms start a mutual business for mutual profits, it is necessary for them to do so with honesty, trust, and dependency on each other. Businesses that are lacking in any part of their business performance could easily utilize coopetition from other businesses that are willing to join them. Importantly, to develop such a relationship, it is important the management of these businesses are willing to partake in coopetition and that they consider the role of open innovation because the organizations join each other with the purpose of open innovation to increase their performances from knowledge and idea sharing idea. In this way, these business firms can enhance their business performance with the help of decision-making, open innovation, and coopetition. On the other hand, according to Zhang et al. (2018), the businesses that fail to join other firms in coopetition are backward, and most are not capable enhancing their market share or revenue. For such business entities, it is necessary to join the competitors based on coopetition to enhance the sharing of external experience, knowledge, and open innovation to increase the business performance in China.

## Conclusion

This study has discussed the mediating role of open innovation and decision-making to improve the business performance while in coopetition among the SMEs of China. Many researchers have attempted to determine if there is a direct link between coopetition and firm performance in the past. However, all of these conclusions are vague and inconclusive, with some reporting a positive association and others declaring a negative relationship. Some researchers have indicated that this relationship may be mediated by other variables that have an indirect impact on it and that these mediators should be investigated to better understand the impact of coopetition on firm performance. Using two variables (decision-making and open innovation) as parallel mediators to describe the relationship between coopetition and firm performance, we were able to close this study gap. Using data from 230 participants, this study investigated the mediating effects of open innovation and decision-making in the relationship between coopetition and firm performance in this research. It is observed that coopetition has an influence on the firm performance, open innovation, and decision-making, and the findings confirm all of the assumptions that were put out in the study. Coopetition, decision-making, and open innovation are all linked with firm performance and both mediators show mediation between IV and DV. All of these findings are congruent with those of prior research. Coopetition has a favorable influence on decision-making and open innovation is responsible for mediating the impact of coopetition on firm performance in a significant way. A direct link between coopetition and firm performance, which had not been previously investigated, has been discovered; in addition, we discovered that decision-making has a favorable impact on firm performance when coopetition is present. Considering the mediating function of decision-making as well as open innovation in the relationship between coopetition and firm performance is a critical step in understanding the costs and advantages of a single corporate management structure for a corporation.

To summarize the study’s findings, this study elaborates on the research’s results and contributions and will emphasize the relationship between coopetition, which will aid in increasing firm performance. Relationships have been studied in an experimental setting, which included mediating variables, to evaluate both intrinsically and extrinsically using bridge factors. Using these factors to compare and combine coopetition studies, researchers may gain a better understanding of the relationships that exist within and between them. Researchers also analyzed the complications, significant linkages, and causal relationships and organized them into an overall view of the mechanisms of coopetition. Related to the coopetition results, firms’ capacity to manage coopetition and balance the contradictory market throughout execution has a direct effect on the results. Once implemented successfully, the advantages of coopetition are many, and they consist of both concrete and intangible benefits, such as invention, joint education, and training. Furthermore, despite the numerous favorable results of coopetition, it must be acknowledged that coopetition is not always helpful and has some negative side effects. Finally, our research demonstrates how the influence of coopetition on firm performance is currently evolving in the real world Coopetition for innovation continues to be a prominent issue of discussion, as research pushes toward the examination of new themes such as the roles of global competition and capabilities, as well as a more comprehensive understanding of coopetition results. Cooperative research dimensions let us link and integrate a fragmented collection of research results into one coherent framework, which helps us contribute to the coopetition literature. Cooperative research dimensions of the model demonstrate how disparate results and aspects relate to one another and fit together, resulting in the development of a cohesive view of a research landscape that was previously dispersed and disjointed. Significantly, this study is useful for emerging as well as already working businesses.

## Future Directions

The limitations of this study are addressed by future research. It was just the mediating function of decision-making and open innovation on the relationship between coopetition and firm performance that was examined in the present research. Franchise development and diversification were not included in the analysis since they are critical firm strategies. Due to the potential for these aspects to explain the effect of coopetition on firm performance, future research may explore these variables as mediators or moderators in firm performance. Future research may explore these variables as mediators or moderators in firm performance as the role of mutual benefits and supply chain is to improve the business performance with coopetition, because there are limited studies addressing the role of the supply chain in coopetition.

## Data Availability Statement

The original contributions presented in this study are included in the article/supplementary material, further inquiries can be directed to the corresponding author.

## Ethics Statement

The studies involving human participants were reviewed and approved by the Zhejiang Wanli University, China. The patients/participants provided their written informed consent to participate in this study. The study was conducted in accordance with the Declaration of Helsinki.

## Author contributions

YC and LX: conceived and designed the concept. KS and CC: data collection. AA: wrote the manuscript. All authors read and agreed to the published version of the manuscript.

## Conflict of Interest

The authors declare that the research was conducted in the absence of any commercial or financial relationships that could be construed as a potential conflict of interest.

## Publisher’s Note

All claims expressed in this article are solely those of the authors and do not necessarily represent those of their affiliated organizations, or those of the publisher, the editors and the reviewers. Any product that may be evaluated in this article, or claim that may be made by its manufacturer, is not guaranteed or endorsed by the publisher.
